# Discrimination of infectious hepatitis A virus and rotavirus by combining dyes and surfactants with RT-qPCR

**DOI:** 10.1186/1471-2180-13-216

**Published:** 2013-10-01

**Authors:** Coralie Coudray-Meunier, Audrey Fraisse, Sandra Martin-Latil, Laurent Guillier, Sylvie Perelle

**Affiliations:** 1 ANSES, Food Safety Laboratory, Food and Water Virology Unit, 23 Avenue du Général de Gaulle, 94706 Maisons-Alfort cedex, France; 2 ANSES, Food Safety Laboratory, Modelling of Bacterial Behaviour Unit, 23 Avenue du Général de Gaulle, 94706 Maisons-Alfort cedex, France

**Keywords:** Propidium monoazide, Ethidium monoazide, Surfactant, RT-qPCR, Hepatitis A virus, Rotavirus, Thermal inactivation, Infectivity

## Abstract

**Background:**

Human enteric viruses are major agents of foodborne diseases. Because of the absence of a reliable cell culture method for most of the enteric viruses involved in outbreaks, real-time reverse transcriptase PCR is now widely used for the detection of RNA viruses in food samples. However this approach detects viral nucleic acids of both infectious and non infectious viruses, which limits the impact of conclusions with regard to public health concern. The aim of the study was to develop a method to discriminate between infectious and non-infectious particles of hepatitis A virus (HAV) and two strains of rotavirus (RV) following thermal inactivation by using intercalating dyes combined with RT-qPCR.

**Results:**

Once the binding of propidium monoazide (PMA) or ethidium monoazide (EMA) was shown to be effective on the viral ssRNA of HAV and dsRNA of two strains of RV (SA11 and Wa), their use in conjunction with three surfactants (IGEPAL CA-630, Tween 20, Triton X-100) prior to RT-qPCR assays was evaluated to quantify the infectious particles remaining following heat treatment. The most promising conditions were EMA (20 μM) and IGEPAL CA-630 (0.5%) for HAV, EMA (20 μM) for RV (WA) and PMA (50 μM) for RV (SA11). The effectiveness of the pre-treatment RT-qPCR developed for each virus was evaluated with three RT-qPCR assays (A, B, C) during thermal inactivation kinetics (at 37°C, 68 C, 72°C, 80°C) through comparison with data obtained by RT-qPCR and by infectious titration in cell culture. At 37°C, the quantity of virus (RV, HAV) remained constant regardless of the method used. The genomic titers following heat treatment at 68°C to 80°C became similar to the infectious titers only when a pre-treatment RT-qPCR was used. Moreover, the most effective decrease was obtained by RT-qPCR assay A or B for HAV and RT-qPCR assay B or C for RV.

**Conclusions:**

We concluded that effectiveness of the pre-treatment RT-qPCR is influenced by the viral target and by the choice of the RT-qPCR assay. Currently, it would be appropriate to further develop this approach under specific conditions of inactivation for the identification of infectious viruses in food and environmental samples.

## Background

Food-borne enteric viruses, particularly human noroviruses (NoV), rotaviruses (RV) and hepatitis A virus (HAV), constitute a serious public health concern, since they are responsible for the vast majority of cases of non-bacterial gastroenteritis and infectious hepatitis, which may occasionally be fatal [1,2]. These viruses are able to replicate in the human gastro-intestinal tract and are dispersed by shedding in high concentrations into the stools. The stability of these viruses with regard to several physical conditions such as pH and temperature, and their resistance to different treatment systems, contribute significantly to their persistence in the environment [3,4]. Transmission of these viruses occurs by the faecal-oral route, primarily through direct person-to-person contact, but they are also efficiently transmitted by ingestion of contaminated drinking water or contaminated foods such as raw shellfish, fresh fruits and vegetables [5].

To ensure the safety of these products, the development of sensitive, reliable techniques for the detection of enteric viruses in food and water samples is helpful. The cell culture system is the gold standard to examine the infectivity of the isolated viruses. Currently, detection of the main enteric viruses on the basis of their infectivity is complicated by the absence of a reliable cell culture method and the low contamination levels of food samples. Thus, molecular methods have been developed for the rapid detection of viral contamination of foods [6,7]. In 2004, the European Committee for Standardisation (CEN) asked a technical advisory group (TAG4) to develop standard methods (qualitative / quantitative) for the detection of norovirus and HAV in foodstuffs. Standard methods have recently been elaborated for a range of risk foods including bottled water, soft fruits and vegetables. The CEN/ISO/TS 15216 standard was published in the first half of 2013 and within a year these proposed protocols will be validated and then published as ISO or CEN standard methods [8]. All these methods are based on a final detection of the viral genome using real-time reverse transcriptase PCR (RT-qPCR), used for its sensitivity, specificity, speed and ability to deliver quantitative data. However, this approach detects the viral nucleic acids of both infectious and non-infectious viruses.

Therefore, it is important to develop and evaluate simple and efficient tools which make it possible to overcome the limitations of the traditional cell culture and PCR assays [9]. An approach based on an enzymatic treatment with RNAse combined with a proteinase K treatment was found to be successful in some cases in distinguishing between infectious and non-infectious viruses [10-12]. For bacteria, a relatively recent approach is the treatment of samples with the DNA-intercalating dyes ethidium monoazide (EMA) or propidium monoazide (PMA) [13-17]. EMA and PMA are closely related DNA intercalating dyes with a photo-inducible azide group that covalently cross-link to DNA through visible-light photoactivation. PMA has the advantage of being more selective than EMA for dead cells as it is more membrane-impermeant [18]. Recently, promising PMA / EMA treatments have also been tested for distinguishing between infectious and non-infectious RNA viruses [19,20]. A study concluded that PMA-RT-PCR assays that include pretreatment of enteroviruses and noroviruses with PMA prior to RT-PCR enable rapid differentiation between infectious and non-infectious enteric viruses when the virus particles are inactivated by heating at 72°C or 37°C or by using hypochlorite. However, unlike poliovirus, PMA treatment did not affect detection of heat-inactivated Norwalk virus by quantitative RT-PCR [21]. Another study found that EMA did not distinguish between infectious and non-infectious avian influenza virus particles [22]. Sánchez et al. [23] showed that PMA treatment previous to RT-qPCR detection is a promising alternative for assessing HAV infectivity.

The usefulness of EMA or PMA for distinguishing between infectious and non-infectious RV and HAV was investigated. Both viruses were chosen for their cultivability and their differences in genomic organization. RV, the leading cause of severe dehydrating diarrhea in infants and young children worldwide, are non-enveloped viruses that possess a genome with 11 segments of double-stranded RNA contained in a triple-layered protein capsid and belong to the *Reoviridae*. Hepatitis A virus (HAV) infection is the leading worldwide cause of acute viral hepatitis. HAV is a positive single-stranded non-enveloped RNA virus classified in the *Hepatovirus* genus of the *Picornaviridae* family.

The purpose of this study was to develop a method based on pre-treatment-RT-qPCR assays in order to discriminate between infectious and non-infectious viruses (HAV, RV) following thermal inactivation. To this end, the binding of EMA and PMA to RV and HAV RNA was investigated. Then, a pre-treatment based on “PMA or EMA +/− surfactant RT-qPCR” was optimized for each virus. Finally, this method was applied to establish viral thermal inactivation kinetics through three RT-qPCR assays.

## Results

### Standard curves of RT-qPCR assays on viral RNA

Linear regression analyses were performed by plotting the cycle threshold (Ct) values against the logarithm of the PFU of HAV or TCID_50_ (50% tissue culture infectious dose) of RV (SA11 and Wa) with RT-qPCR assays A, B and C corresponding to the RNA target. The mean parameters of the standard curves were as follows: standard curves respectively obtained with HAV assays A, B and C showed efficiencies of 100.00%, 95.93%, and 104.83% and regression coefficients of 0.999, 0.997, 0.996; standard curves respectively obtained with RV assays A, B and C showed efficiencies of 90.93%, 94.03%, and 94.23% and regression coefficients of 0.993, 0.986, 0.976 with Wa; standard curves respectively obtained with RV assays A, B and C showed efficiencies of 78.83%, 76.53%, and 85.50% and regression coefficients of 0.989, 0.984, 0.989 with SA11.

### Evaluation of dyes-RT-qPCR assays on viral RNA

The first experiments studied the efficiency of PMA and EMA treatments to bind the viral RNA in order to avoid its detection (RV, HAV) using RT-qPCR assays A and the potential inhibitory effects of the dyes on RT-qPCR amplification (Table 1). Viral RNA was treated with dye concentrations ranging from 10 to 200 μM without photoactivation and then subjected to RT-qPCR to determine if residual dyes can be inhibitors for RT-qPCR (Table 1A). In the lowest PMA concentration (10 μM), an inhibitory effect on RT-qPCR detection was only found for RV RNA (Wa and SA11) (respectively a decrease of - 0.87 log_10_ and - 1.47 log_10_ of detected RNA). With 20 μM of PMA, an inhibitory effect on RT-qPCR was also found for HAV RNA (− 1.59 log_10_). PMA concentrations ranging from 50 μM to 200 μM were able to totally inhibit the RT-qPCR amplification of viral RNA. Inhibitory effects of EMA were found from 20 μM on RV (Wa) (− 1.18 log_10_), and from 50 μM on HAV (− 0.99 log_10_). Higher concentrations of EMA totally inhibited RT-qPCR assays on HAV and RV (Wa) viral RNA. Inversely, no inhibitory effect of any of the EMA concentrations tested was observed with RV (SA11) RNA. The efficacy of the purification of excess dye in treated RNA samples using the QIAquick PCR purification kit was tested to avoid inhibitory effects on RT-qPCR amplification (Table 1B). Purification by QIA-quick showed effective recovery with a decrease in viral titer ≤ − 0.49 log_10_ with RNA samples not treated with monoazide. The purification step was found to be effective in removing residual dye, except for RV (SA11) RNA samples which were treated with PMA ranging from 50 to 200 μM.

**Table 1:** Binding of dyes to purified viral RNA

**[Dye] μM**	**HAV**		**RV (Wa)**		**RV (SA11)**	
	**PMA**	**EMA**	**PMA**	**EMA**	**PMA**	**EMA**
A						
10	−0.09 ± 0.11	−0.12 ± 0.09	−0.87 ± 0.30	−0.52 ± 0.19	−1.47 ± 1.27	−0.41 ± 0.27
20	−1.59 ± 0.74	−0.21 ± 0.27	−1.87	−1.18 ± 0.46	−2.51 ± 0.69	−0.31 ± 0.31
50	< LOD	−0.99 ± 0.51	< LOD	< LOD	< LOD	−0.47 ± 0.15
100	< LOD	< LOD	< LOD	< LOD	< LOD	−0.44 ± 0.47
200	< LOD	< LOD	< LOD	< LOD	< LOD	−0.30 ± 0.41
B						
0	−0.33 ± 0.10	−0.33 ± 0.10	−0.49 ± 0.51	−0.49 ± 0.51	−0.07 ± 0.26	−0.07 ± 0.26
10	−0.55 ± 0.13	−0.41 ± 0.26	−0.39 ± 0.11	−0.16 ± 0.06	−0.51 ± 0.16	−0.32 ± 0.32
20	−0.25 ± 0.27	0.37 ± 0.05	−0.27 ± 0.22	−0.37 ± 0.12	−0.68 ± 0.49	−0.28 ± 0.23
50	0.32 ± 0.26	0.43 ± 0.51	−0.34 ± 0.09	−0.23 ± 0.20	−1.60	−0.32 ± 0.23
100	−0.54 ± 0.01	0.03 ± 0.14	−0.38 ± 0.18	0.35 ± 0.24	< LOD	0.52 ± 0.23
200	−0.36 ± 0.13	0.35 ± 0.24	−0.30 ± 0.20	−0.47 ± 0.35	< LOD	−0.34 ± 0.16
C						
0	−0.33 ± 0.10	−0.33 ± 0.10	−0.49 ± 0.51	−0.49 ± 0.51	−0.07 ± 0.26	−0.07 ± 0.26
10	−2.65 ± 0.51	−0.96 ± 0.27	−1.27 ± 0.12	−0.59 ± 0.24	−1.41 ± 0.51	−0.79 ± 0.50
20	−2.27 ± 0.46	−1.08 ± 0.48	−1.33 ± 0.13	−0.07 ± 0.50	−1.48 ± 0.55	−0.64 ± 0.66
50	−3.16 ± 0.77	−1.16 ± 0.21	−1.75 ± 0.11	−0.62 ± 0.38	−2.96 ± 1.38	−1.22 ± 0.67
100	−2.47 ± 0.37	−1.56 ± 0.33	−2.20 ± 0.50	−1.01 ± 0.11	−3.58 ± 0.65	−2.06 ± 1.63
200	−2.91 ± 0.63	−1.53 ± 0.17	−2.52 ± 1.13	−0.99 ± 0.41	−3.02 ± 1.10	−0.63 ± 0.55

Quantification by RT-qPCR assays A of 10^8^ copies of the genome of viral RNA after monoazide treatment without photoactivation (**A**), after monoazide treatment without photoactivation followed by QIA-quick purification (**B**), after monoazide treatment with photoactivation followed by QIA-quick purification (**C**). Mean values ± SD (n=3).

Lastly, optimal PMA / EMA concentrations were determined on viral RNA samples after dye treatment including photoactivation and purification steps. The effects of dye (concentrations of 10 to 200 μM) were determined by measuring the decrease in RNA quantification by RT-qPCR (Table 1C). PMA at 50 μM enabled the highest reduction of the RT-qPCR signal for HAV RNA (− 3.16 log_10_) and PMA at 100 and 200 μM respectively enabled the highest reductions of the RT-qPCR signal for RV (SA11) (− 3.58 log_10_) and RV (Wa) (− 2.52 log_10_). EMA was still found to be less efficient than PMA treatment for all the viral RNA tested. These data showed that PMA and EMA are able to bind to viral RNA upon photoactivation making the RNA unavailable for amplification by RT-qPCR, although excess dye concentrations can inhibit RT-qPCR assays. The effectiveness of PMA and EMA treatments depends on the type of dye, the concentration of the dye and the viral RNA type, although PMA was found to be the most effective dye for the three viral RNA tested.

### Optimization of pretreatment combining dyes and surfactants before RT-qPCR assays for the selective detection of infectious viruses

#### Determination of optimal PMA / EMA concentrations

Table 2 shows the results of experiments conducted with viruses (HAV and RV (Wa, SA11)) to optimize a specific procedure based on dye treatment for selective detection of the viral RNA from infectious viruses using RT-qPCR assays A.

**Table 2:** Influence of dye concentration on viruses

**Titration method**	**Virus**	**Infectious / inactived**	**PMA (μM)**					**EMA (μM)**				
			**5**	**20**	**50**	**75**	**100**	**5**	**20**	**50**	**75**	**100**
RT-qPCR	HAV	Infectious	0.03 ± 0.08	0.02 ± 0.08	−0.03 ± 0.02	−0.08 ± 0.01	−0.02 ± 0.05	−0.10 ± 0.17	−0.04 ± 0.02	−0.07 ± 0.07	−0.05 ± 0.05	−0.09 ± 0.03
		Inactived	−0.88 ± 0.12	−1.01 ± 0.08	−1.06 ± 0.11	−1.13 ± 0.09	−1.14 ± 0.09	−1.24 ± 0.13	−1.75 ± 0.91	−1.31 ± 0.28	−1.25 ± 0.24	−1.17 ± 0.23
	RV (SA11)	Infectious	−0.28 ± 0.38	−0.32 ± 0.44	−0.30 ± 0.33	−0.68 ± 0.41	−0.51 ± 0.28	−0.70 ± 0.12	−0.70 ± 0.30	−0.71 ± 0.08	−0.75 ± 0.09	−0.72 ± 0.09
		Inactived	−1.16 ± 0.68	−1.45 ± 0.78	−1.60 ± 0.57	−1.70 ± 0.40	−1.71 ± 0.50	−1.12 ± 0.31	−1.13 ± 0.19	−1.05 ± 0.33	−1.06 ± 0.24	−1.07 ± 0.07
	RV (Wa)	Infectious	0.05 ± 0.09	−0.38 ± 0.34	−0.63 ± 0.02	−0.62 ± 0.14	−0.52 ± 0.15	−0.19 ± 0.05	−0.50 ± 0.20	−0.96 ± 0.31	−1.12 ± 0.16	−1.15 ± 0.13
		Inactived	−0.24 ± 0.65	−0.62 ± 0.27	−1.00 ± 0.15	−1.44 ± 0.18	−1.45 ± 0.29	−0.52 ± 0.76	−1.51 ± 0.26	−1.81 ± 0.06	−1.72 ± 0.19	−1.48 ± 0.18

Quantification by RT-qPCR assays A after monoazide treatment of 10^5^TCID_50_ of RV (SA11), 10^3^ TCID_50_ of RV (Wa) and 6× 10^4^ PFU of HAV, infectious or inactivated at 80°C for 10 minutes. Mean values ± SD (n=3).

As the first step in exploring the potential of PMA and EMA to detect infectious viruses, HAV, RV (SA11) and RV (Wa) viruses were either inactivated thermally or not, and were subjected to dye concentrations ranged from 5 to 100 μM, photoactivation, RNA extraction and quantification by RT-qPCR (Table 2). The presence of PMA or EMA had no effect on detection of the RNA extracted from infectious HAV regardless of the concentration tested. Similarly, quantification of RNA extracted from PMA-treated infectious RV was not strongly affected by decreases ranging from - 0.05 log_10_ to - 0.63 log_10_ for Wa and from - 0.28 log_10_ to - 0.68 log_10_ for SA11, depending on the PMA concentrations tested. However, quantification of RNA extracted from infectious RV was more strongly affected by EMA treatment, with a decrease between - 0.19 log_10_ and - 1.15 log_10_ for Wa and between - 0.70 log_10_ and - 0.75 log_10_ for SA11, depending on the EMA concentrations tested.

When thermally inactivated viruses were assayed with PMA RT-qPCR, maximum decreases were found for HAV (− 1.06 log_10_ to −1.14 log_10_) and for RV (SA11) (− 1.60 log_10_ to - 1.71 log_10_) with PMA concentrations ranging from 50 μM to 100 μM, and for RV (Wa) (− 1.44 log_10_ and - 1.45 log_10_) with PMA concentrations of 75 μM and 100 μM. When inactivated viruses were assayed with EMA RT-qPCR, maximum decreases were found for HAV (− 1.75 log_10_) with EMA at 20 μM, for RV (SA11) (− 1.13 log_10_) with EMA at 20 μM, and for RV (Wa) (− 1.81 log_10_) with EMA at 50 μM.

The data obtained with all the negative controls were as expected. Treatment by PMA / EMA without photoactivation or with a single exposure of the viruses to light before RNA extraction did not significantly affect the RT-qPCR detection of extracted RNA (data not shown).

The most effective dye concentration for each virus was experimentally chosen by taking into account the effect of the dye concentrations on the inactivated and infectious viruses. In cases where similar data were observed with different dye concentrations, the lowest dye concentration was preferred. Thus, 20 μM of EMA for all viruses, 50 μM of PMA for HAV and RV (SA11) and 75 μM of PMA for RV (Wa) were selected as optimal concentrations.

#### Evaluation of pre-treatments combining dye and surfactant

As a second step, Triton X-100, Tween 20 and IGEPAL CA-630, three widely used nonionic surfactants, were tested for their efficacy in improving the effects of PMA / EMA treatment on viral particles (Table 3).

**Table 3:** Influence of combined dyes and surfactants on viruses

**Titration method**	**Virus**	**Infectious / inactived**	**Dye**	**Triton ×100**			**Tween 20**			**IGEPAL CA-630**		
				**0.1%**	**0.5%**	**1%**	**0.1%**	**0.5%**	**1%**	**0.1%**	**0.5%**	**1%**
RT-qPCR	HAV	Infectious	EMA (20 μM)	0.03 ± 0.07	−0.06 ± 0.06	−0.05 ± 0.05	−0.02 ± 0.09	−0.07 ± 0.09	−0.02 ± 0.06	0.02 ± 0.13	−0.02 ± 0.05	−0.04 ± 0.09
		Inactived		−2.42 ± 0.04	−2.52 ± 0.10	−2.48 ± 0.01	−1.70 ± 0.05	−1.88 ± 0.29	−1.89 ± 0.08	−2.23 ± 0.41	−2.68 ± 0.01	−2.42 ± 0.07
		Infectious	PMA (50 μM)	−0.07 ± 0.02	−0.07 ± 0.02	0.00 ± 0.02	−0.05 ± 0.06	−0.12 ± 0.07	−0.09 ± 0.09	−0.06 ± 0.08	−0.04 ± 0.05	−0.07 ± 0.10
		Inactived		−2.34 ± 0.27	−2.49 ± 0.25	−2.51 ± 0.23	−1.74 ± 0.07	−1.70 ±0.09	−1.70 ± 0.11	−2.42 ± 0.27	−2.49 ± 0.34	−2.34 ± 0.19
	RV (SA11)	Infectious	EMA (20 μM)	−0.80 ± 0.10	−0.77 ± 0.08	0.47 ± 0.11	0.75 ± 0.14	−0.72 ± 0.07	−0.68 ± 0.09	−0.79 ± 0.07	−0.47 ± 0.09	−0.71 ± 0.09
		Inactived		−1.66 ± 0.09	1.43 ± 0.15	−1.14 ± 0.28	−1.18 ± 0.17	−1.89 ± 0.77	−1.28 ± 0.20	−1.30 ± 0.13	−1.28 ± 0.30	−0.81 ± 0.27
		Infectious	PMA (50 μM)	−0.74 ± 0.15	−0.77 ± 0.16	−0.91 ± 0.20	0.80 ± 0.11	−0.76 ± 0.20	−0.80 ± 0.20	−0.72 ± 0.14	0.71 ± 0.23	−0.81 ± 0.18
		Inactived		−1.34 ± 0.18	−1.29 ± 0.13	−1.33 ± 0.22	−1.30 ± 0.15	−1.39 ± 0.16	−1.31 ± 0.49	−1.31 ± 0.27	−1.35 ± 0.25	−1.14 ± 0.39
	RV (Wa)	Infectious	EMA (20 μM)	−0.39 ± 0.07	−0.24 ± 0.13	−0.15 ± 0.10	−0.41 ± 0.06	−0.13 ± 0.13	−0.37 ± 0.17	−0.28 ± 0.22	−0.21 ± 0.02	0.36 ± 0.13
		Inactived		−1.21 ± 0.14	−0.68 ± 0.12	−0.40 ± 0.16	−1.01 ± 0.19	−0.88 ± 0.15	−0.58 ± 0.16	−0.82 ± 0.43	−0.71 ± 0.08	−0.14 ± 0.13
		Infectious	PMA (75 μM)	−0.57 ± 0.14	−0.61 ± 0.18	−0.61 ± 0.13	−0.58 ± 0.15	−0.58 ± 0.11	−0.64 ± 0.14	−0.60 ± 0.16	−0.58 ± 0.15	−0.70 ± 0.16
		Inactived		−1.23 ± 0.08	−1.11 ± 0.04	−1.20 ± 0.18	−1.21 ± 0.08	−1.15 ± 0.09	−1.15 ± 0.17	−1.21 ± 0.08	−1.15 ± 0.18	−1.23 ± 0.08
Cell culture	HAV	Infectious	None	0.09 ± 0.22	−0.03 ± 0.17	0.02 ± 0.21	0.11 ± 0.11	0.16 ± 0.06	0.04 ± 0.25	0.06 ± 0.17	−0.01 ± 0.01	0.14 ± 0.09

Quantification by RT-qPCR assays A after monoazide treatment combined with surfactants (Triton ×100, Tween-20, IGEPAL CA-630) of 10^5^ TCID_50_ of RV (SA11), 10^3^ TCID_50_ of RV (Wa) and 6× 10^4^ PFU of HAV, infectious or inactivated at 80°C for 10 minutes, and titration by cell culture of 6× 10^4^ PFU of infectious HAV treated with surfactants. Mean values ± SD (n=3).

Beforehand, we attempted to evaluate surfactant toxicity towards the infectivity of HAV and rotavirus strains, which would preclude their use as a discriminatory treatment. One set of HAV viral samples receiving surfactants was compared to untreated HAV viral samples by titration with cell culture. None of the surfactant treatments significantly reduced the initial HAV titer (≤ 0.20 log_10_), which argues in favor of the use of a dye-surfactant pre-treatment. It was not possible to measure the toxicity of surfactants to RV strains (Wa and SA11) because all surfactant doses affected the MA104 cells in culture (data not shown).

The previously selected optimal dye concentration for each virus (20 μM of EMA for all viruses, 50 μM of PMA for HAV and RV (SA11) and 75 μM of PMA for RV (Wa)) were tested in association with three concentrations of three surfactants.

When inactivated HAV was assayed, Tween 20 only very slightly increased the efficacy of PMA (50 μM) (<− 0.7 log_10_) and did not increase the efficacy of EMA (20 μM) pretreatments. The pretreatments of inactivated HAV associating PMA (50 μM) with IGEPAL CA-630 or Triton ×100 improved the processing regardless of the concentration of surfactant tested. Indeed, the logarithmic reductions of RNA detected by RT-qPCR were included between - 2.34 log_10_ and - 2.49 log_10_ which was higher than the reduction of 1.06 log_10_ obtained with PMA treatment at 50 μM. Similarly, the processing of inactivated HAV associating EMA (20 μM) with IGEPAL CA-630 or Triton ×100, regardless of the concentration of surfactant tested, enhanced the efficacy of the processing. Indeed, the logarithmic reductions of RNA detected by RT-qPCR were included between - 2.23 log_10_ and - 2.68 log_10_ which was higher than the reduction of 1.75 log_10_ obtained with EMA treatment at 20 μM. Finally, the treatment of HAV by the most promising IGEPAL CA-630 (0.5%) without monoazide or photoactivation before RNA extraction did not affect RT-qPCR detection of extracted RNA, which argues in favor of the use of a dye-surfactant pre-treatment (data not shown).

When inactivated RV (SA11) was assayed, the efficacy of the processing with PMA (50 μM) was always slightly higher without surfactant. When inactivated RV (SA-11) was assayed with EMA and surfactants, the highest improvement was found with Tween 20 (0.5%) leading to an increase of reduction of RNA detected by RT-qPCR of −0.76 log_10_ compared with treatment with EMA at 20 μM. However, the pre-treatment based on EMA also seemed to affect RNA detection from infectious RV (SA11) (− 0.72 log_10_) more than the pre-treatment based on PMA (− 0.30 log_10_). When inactivated RV (Wa) was assayed, none of the tested surfactants increased the efficacy of the dye pretreatments.

By taking into account all these data, we selected pre-treatments with EMA (20 μM) and IGEPAL CA-630 (0.5%) for HAV, with EMA (20 μM) for RV (Wa) and PMA (50 μM) for RV (SA11) for their high efficiencies. Since different incubation times (30 min, 2 h, overnight) did not change the selected pre-treatment efficiencies (data not shown), an incubation time of 2 h was selected for the following studies.

### Kinetic thermal viral inactivation curves and impact of RT-qPCR assays

**Figure 1:**
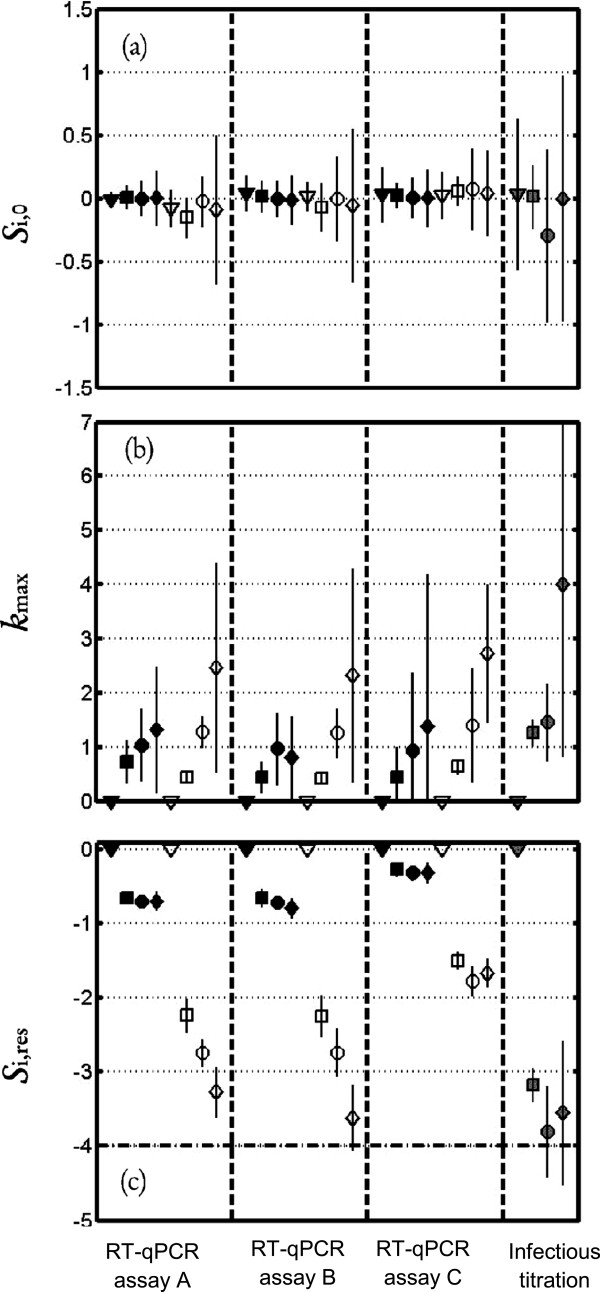
**Thermal inactivation kinetics of HAV.** Thermal Inactivation kinetics of HAV **(a,b,c)**, expressed with the log-linear + tail model: *log*_10_(*S*_*i*_(*t*)) = *log*_10_((*S*_*i*,0_ − *S*_*i*,*res*_) · *exp*(−*k*_max_ · *t*) + *S*_*i*,*res*_) (Equation 2). Plots of the estimated parameters for Equation 2 and the corresponding 95% asymptotic confidence intervals for HAV. **(a)***S*_i,0_; **(b)***k*_max_; **(c)***S*_i,res_. The results obtained at 37°C, 68°C, 72°C and 80°C are indicated by ▼, ■, ● and ◆ respectively. Symbol shaded in gray indicates data obtained with cell culture method, symbol in black indicates RT-qPCR and open symbol represents RT-qPCR with pre-treatment. (- -) Limit of quantification.

**Figure 2:**
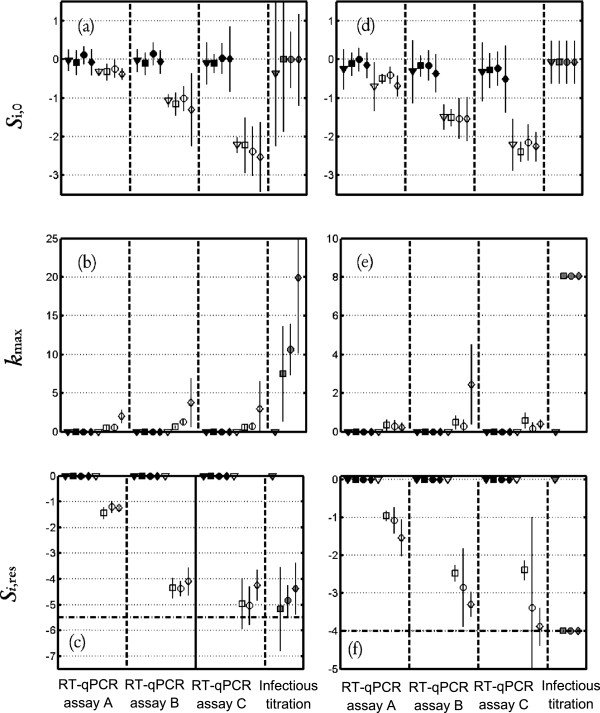
**Thermal inactivation kinetics of RV.** Thermal Inactivation kinetics of RV (Wa) **(a,b,c)** and RV (SA11) **(d,e,f)** expressed with the log-linear + tail model: *log*_10_(*S*_*i*_(*t*)) = *log*_10_((*S*_*i*,0_ − *S*_*i*,*res*_) · *exp*(−*k*_max_ · *t*) + *S*_*i*,*res*_) (Equation 2). Plots of the estimated parameters for Equation 2 and the corresponding 95% asymptotic confidence intervals for Wa and SA11 respectively. **(a, d)** Si,0; **(b, e)** kmax; **(c, f)***S*_i,res_. The results obtained at 37°C, 68°C, 72°C and 80°C are indicated by ▼, ■, ● and ◆ respectively. Symbol shaded in gray indicates data obtained with cell culture method, symbol in black by RT-qPCR and open symbol represents RT-qPCR with pre-treatment. (- -) Limit of quantification.

The heat sensitivity of HAV and RV (Wa, SA11) at four temperatures (37°C, 68°C, 72°C, 80°C) was analyzed by kinetic evaluation of the loss of infectivity in cell culture compared with the loss of genomic titer in RT-qPCR assays with or without pre-treatment. Stable secondary structures may facilitate the covalent binding of PMA / EMA to viral RNA rendering the RNA undetectable by RT-qPCR. Moreover, amplicon length may influence the effectiveness of these assays. Three RT-qPCR assays were assayed for each viral target to explore the impact of the amplified genomic region on the success of the pre-treatment-RT-qPCR assays in detecting the infectious viruses. The log_10_ reduction detection limits of the cell culture technique were −4 log_10_ PFU of HAV, -5.5 log_10_ TCID_50_ of RV (Wa) and −3.5 log_10_ TCID_50_ of RV (SA11). For describing all the inactivation curves, the log-linear + tail model was found to be the most appropriate. Figures 1 and 2 show the values of the parameters of Equation (2) that characterized the fate of the HAV and RV strain levels respectively according to the four different temperatures, and to the three methods of quantification of the virus titer, i.e. RT-qPCR and pre-treatment RT-qPCR depending on the three different RT-qPCR assays used and the infectious titer.

For HAV, the values of S_i,0_ were not different from zero, which means that the EMA IGEPAL CA-630 treatment did not affect virus quantification with regard to the RT-qPCR method. At 37°C, the level of HAV remained constant regardless of the method used. For other temperatures, *k*_max_, which is the inactivation rate, increased with temperature. Using a Bigelow-type model, relating *D*-value to temperature, this increase corresponds to z-values ranging from 17°C to 22°C for EMA-IGEPAL CA-630 -RT-qPCR and infectious titration methods and of 44°C for the RT-qPCR method, regardless of the RT-qPCR assay. Confidence intervals of *S*_i,res_ indicate that the fraction of virus that can survive thermal treatment differs depending on the titration method used and the temperature. With EMA-IGEPAL CA-630 - RT-qPCR and RT-qPCR assay C, the *S*_2,res_ value is approximately −1.6 log_10,_ which means that 1 virus out of 40 is quantifiable after 20 min of treatment regardless of the temperature. With EMA-IGEPAL CA-630 - RT-qPCR and RT-qPCR assay A or B, between 1 virus out 200 and 1 virus out of 6000 is still quantifiable after treatment at 68°C and 80°C (with *S*_2,res_ ranged between −2.3 log_10_ and −3.8 log_10_). For RT-qPCR, *S*_1,res_ are much higher than *S*_2,res_, but the difference between RT-qPCR assays A and B and RT-qPCR assay C was also observed for RT-qPCR. For the infectious titration method, *S*_3,res_ is around −3.5 log_10,_ close to the values obtained with EMA-IGEPAL CA-630 - RT-qPCR associated with RT-qPCR assays A and B.

For RV strains, the values of S_2,0_ were lower than zero, which means that the EMA / PMA treatment affected virus quantification with regard to the RT-qPCR method. Indeed, the reduction of the concentration of infectious virus due to the monoazide pre-treatment was about of −0.5 log_10_ by using RT-qPCR assay A and is ranged from −1.2 log_10_ to −2.5 log_10_ by using RT-qPCR assays B and C. These reduction levels were the same for both RV strains. At 37°C, the level of RV strains remained constant regardless of the method used. At 68°C, 72°C, and 80°C, the genomic titer of the RV strains was found to be constant by using the RT-qPCR method regardless of the RT-qPCR assay tested. The *S*_i,res_ confidence intervals indicate that the fraction of virus that can survive thermal treatment differs depending on the titration method used.

For the Wa RV strain, with EMA-RT-qPCR and RT-qPCR assay A, the *S*_2,res_ value was approximately −1.3 log_10_ which means 1 virus out of 20 was quantifiable after 20 min of treatment regardless of the temperature. With EMA-RT-qPCR and RT-qPCR assays B or C, between 1 virus out of 10^4^ and 1 virus out of 10^5^, was still quantifiable after treatment at 68°C, 72°C or 80°C (i.e. *S*_2,res_ ranged between −4 log_10_ and −5 log_10_). The S_3,res_ values obtained with the infectious titration method were similar to the *S*_2,res_ values of RT-qPCR assays B and C.

For the SA11 RV strain, with PMA-RT-qPCR and RT-qPCR assay A, *S*_2,res_ value is approximately −1.2 log_10_. With RT-qPCR assays B and C, *S*_2,res_ ranged from −2.4 log_10_ and −3.9 log_10_. The value of *S*_2,res_ with these RT-qPCR assays decreased significantly when the temperature of treatment increases. S_3,res_ values cannot be estimated as inactivation after 1 minute of treatment for 68°C, 72°C or 80°C was higher than the LOQ.

For SA11, the *k*_max_ value increased with the temperature. Using a Bigelow-type model, this increase corresponds to z-values ranging from 15°C to 19°C according to the RT-qPCR assays for the PMA-RT-qPCR method and of 28°C for the infectious titration method. For the Wa strain and EMA-RT-qPCR, the large confidence interval observed for *k*_max_ did not make it possible to detect a temperature effect. Very fast inactivation of Wa strain, (after 1 minute of treatment, infectious titers were below the limit of detection (LOD)) only allows to argue that *k*_max_ values were higher than 8.

In conclusion, assays conducted to examine the efficiency of pre-treatment RT-qPCR in minimizing detection signals from thermally-inactivated viruses were dependent on virus species, on the temperature of inactivation and on the RT-qPCR assays.

## Discussion and conclusion

Foodborne viruses have emerged as a major cause of outbreaks worldwide. Among the factors that affect virus survival, temperature has a great influence on virus stability in food as in any other matrix. Therefore, food industries widely apply temperature as a virus-inactivating factor. Natural or added constituents of food and the virus species may influence the rate of virus inactivation by temperature but higher temperatures provided more pronounced virus decay [24]. The primary model that was found to effectively describe thermal virus inactivation in our study, (i.e. the log-linear + tail primary inactivation model), was similar to the one chosen to describe thermal inactivation of HAV in raspberries [25]. The infectivity of enteric viruses requires the functional integrity of two major components, the capsid and the genome [26]. While quantitative RT-PCR is a specific and sensitive tool for determining the quantities of viral genomes in the environment and food samples, it does not discriminate between infectious viruses and non-infectious viruses that do not pose a threat to health. Moreover, the virus genome was shown to be more resistant than the infectious virus. So, methods which provide information about the infectivity are particularly useful for the detection of enteric viruses and would be an advantage in a public health perspective [27].

Recently, ethidium monoazide (EMA) and propidium monoazide (PMA), which are intercalating dyes, have been used combined with PCR or real-time PCR for the selective detection of viable microorganisms. In this study, monoazides were tested in association with surfactants in order to develop a technique for determining the residual infectivity of thermally inactivated enteric viruses. These assays are based on the penetration of monoazide, potentially facilitated by the action of surfactants, through damaged or compromised capsids and its covalent binding to viral RNA, which makes the genome unavailable for amplification by RT-qPCR.

In this study, it was hypothesized that the PMA / EMA would be able to enter non-infectious RV / HAV viruses and easily bind to the dsRNA of RV and to the highly structured 5’-non coding region (5’-NCR) of HAV ssRNA targeted by the RT-qPCR assays [28]. The pre-treatment RT-qPCR assays with the shortest amplification fragments for RV (87-bp) and HAV (77-bp) did not produce data similar to those obtained by measuring the decrease in the number of infectious particles following heat treatment. By using both longer amplification fragments (313-bp; 352-bp) targeting two different regions of RV dsRNA, data obtained with pretreatment RT-qPCR were very similar suggesting that the targeted region had not influenced the success of the pretreatment RT-qPCR for dsRNA. Similarly, both longer amplification regions for HAV ssRNA (174-bp; 353-bp) provided data suggesting that the stable secondary structures may facilitate covalent binding of monoazide to HAV ssRNA. Thus, the stable secondary structures may facilitate covalent binding of monoazide to viral RNA, rendering the RNA undetectable by RT-qPCR. Besides the targeted genome region, this study also showed the influence of the RT-qPCR assays in terms of length of amplicons for three viruses. Other studies have shown the influence of amplification length on the degree of PCR suppression by monoazide treatment in dead cells [29-31].

The HAV capsid is composed of the structural proteins VP1, VP2, VP3, and possibly VP4, encoded in the P1 region of the genome [32]. Cell culture-derived rotavirus preparations contain a mixture of double-layered particles (DLPs) and triple-layered particles (TLPs). The innermost layer of the rotavirus particle is made up of the core protein VP2, the middle layer is composed entirely of VP6, and the outermost layer of RV is composed of two proteins, VP4 and VP7 [33]. VP4 forms spikes that extend outwards from the surface of the virus and which have been linked to a variety of functions, including initial attachment of the virus to the cell membrane and penetration into the cell by the virion [34]. Indeed, the capsids structures may explain the differences of efficacy of thermal inactivation and of the penetration of monoazide. The presence of monoazide did not affect the measurement of HAV, but it slightly affected the measurement of both rotavirus strains. This effect appeared to be variable (between 0.5 log_10_ and 2.5 log_10_) depending on the RT-qPCR assays and therefore not always an impediment to the use of monoazide pre-treatment for RV. Nevertheless, this monoazide effect seems to be dependent on the virus type and should be evaluated to develop this approach with other viruses.

There is still very little development of monoazide RT-qPCR methods for determining the infectiosity of enteric viruses. Among the few studies reported in the literature, Sánchez et al. [23] found that PMA treatment at 50 μM was significantly more effective than RNase treatment for differentiating infectious and thermally-inactivated HAV (99°C for 5 min), with HAV titers reduced by more than 2.4 log_10_. The EMA – IGEPAL CA-630 - RT-qPCR assay developed in this study was significantly more effective for differentiating infectious and thermally-inactivated HAV with HAV titers reduced by more than 3 log_10_ and 3.8 log_10_ respectively with the RT-qPCR assays A and B after 5 min at 80°C. Z values observed in the present study when infectious titration or pretreatment-RT-qPCR methods were used are consistent with those observed in the meta-analysis of inactivation of enteric viruses in food and water carried out by Bertrand et al. [24]. Nevertheless, when high inactivation temperatures were applied, clearer discriminations between infectious and non-infectious viruses were consistently observed with pre-treatment-RT-qPCR assays. Thus, the procedures reported in the present study provide limits that are comparable to those determined by others [19,20,22]. As the pre-enzymatic treatment-PCR approach, monoazide RT-qPCR depend mainly on capsid integrity as the criterion for infectivity, and this could be one of the drawbacks of this technique since virus inactivation may take place by other means than particle disruption [9].

Optimization of EMA or PMA concentration and the choice of the RT-qPCR assay, as well as the addition of a complementary treatment to enhance the penetration of monoazide into the slightly-damaged capsid may lead to more effective monoazide treatment. This study showed that surfactants may be useful to improve monoazide-RT-qPCR assays for HAV but not for RV.

In conclusion, the lack of information about infectious risk makes it necessary to evaluate new means of preventing a positive RT-qPCR signal in the absence of infectious virus. The pre-treatment of enteric viruses with monoazide alone or in conjunction with other capsid-disrupting aids prior to RT-qPCR may be optimized to obtain rapid differentiation between infectious and non-infectious viruses. This approach can potentially be used with all non-culturable and difficult to culture viruses but must be estimated with regard to the specific conditions of inactivation. Currently, it seems relevant to develop this approach for the identification of infectious viruses in food and environmental samples. However the potential multiple sources of inactivation, such as UVs, storing conditions, temperature, etc., could lead to changes in capsid protein conformation without compromising capsid integrity [9]. This is why it may be necessary to adapt and evaluate the dye treatment according to the inactivation type. Moreover, the efficacy of pre-treatment RT-qPCR assays could be affected by the types of samples (various food and environmental samples) and should be characterized in order to be developed further. Therefore, this new approach could be very useful for evaluating the susceptibility of non-culturable enteric viruses (e.g. norovirus, HAV, HEV) to specific inactivation / disinfection techniques or food processing strategies and could be an alternative to studies using culturable surrogate viruses that differ in structure, function and comportment.

## Methods

### Viruses and cells

HAV strain HM175/18f, clone B (VR-1402) was obtained from the American Type Culture Collection (ATCC). This clone replicates rapidly and has cytopathic effects in cell culture [35]. HAV stock was produced by propagation in foetal rhesus monkey kidney (FRhK-4) cells (ATCC, CRL-1688) [36] and titrated by plaque assay [37]. Results were expressed in plaque-forming units/mL (PFU/mL) and HAV stock contained 10^7^ PFU/mL.

Rotavirus strains SA11 (simian rotavirus A) and Wa (human rotavirus) were obtained from the Pasteur Institute (Paris, France) and were propagated in MA-104 rhesus monkey epithelial cell line (ATCC CRL-2378). MA-104 cells were grown in Minimum Essential Medium - Glutamax™ (MEM), 1% non-essential amino acids, 10% foetal bovine serum and 0.5% penicillin-streptomycin (Life Technologies, France). Cells were incubated at 37°C in an atmosphere containing 5% CO_2_ and grown to sub-confluence. Rotavirus viral stock solutions consisted of an infected cell culture supernatant. Infected cells were frozen and thawed once and then clarified using low-speed centrifugation (6000 × g) at 4°C to remove residual debris. The supernatant of SA11 contained 10^7^ TCID_50_ / mL. The supernatant containing Wa was then ultracentrifugated at 151,000 ×g for 1 h at 4°C to obtain a higher viral titer. The pellet was resuspended in PBS to obtain a Wa stock containing 10^5^ TCID_50_ / mL. Both virus stocks were divided into aliquots and stored at −80°C. For the infectivity assay, sub-confluent MA-104 cells seeded in 96-well plates were washed twice with MEM. Samples were trypsin-activated for 30 min at 37°C, and then added to MA-104 cells. Plates were incubated 3 days at 37°C. Infectious titers of RV were expressed as TCID_50_/mL, according to the Kärber method.

### RNA purification of Rotaviruses and HAV

HAV and RV RNA stocks were produced from infected cell culture supernatants. They were centrifugated at 4,000 g for 30 minutes at 4°C and then the supernatants were ultracentrifugated at 25,000 g for 25 min at 4°C. Finally, supernatants were ultracentrifugated at 151,000 g for 50 min at 4°C and the pellets were suspended in aliquots of 0.7 mL of 1× PBS and incubated overnight at 4°C before virus titration. The viral stocks were then vortexed for about 10 s before RNA extraction. Volumes of 350 μL were supplemented with NucliSens® easyMAG™ lysis buffer (BioMérieux) up to 3 mL and subjected to the NucliSens® easyMAG™ platform for RNA extraction by the “off-board Specific A protocol” according to the manufacturer’s instructions. Lastly, nucleic acids were eluted in 70 μL of elution buffer and pooled to obtain a homogenized RNA stock. To avoid contamination of cellular DNA from the HAV and RV RNA stocks, the samples were treated with the Turbo DNase free-kit (Life Technologies) according to the manufacturer’s instructions. The purified RNA stocks were quantified by measuring absorbance at 260 / 280 nm with a Nanodrop ND-100 (Thermoscientific, France) and the free software available on the “http://endmemo.com/bio/dnacopynum.php” website. All viral RNA stocks (from HAV, SA11 and Wa) containing 10^9^ copies / μL were aliquoted and stored at - 80°C.

### Propidium monoazide (PMA), ethidium monoazide (EMA)

PMA (phenanthridium, 3-amino-8-azido-5-[3-(diethylmethylammonio)propyl]-6-phenyl dichloride) was purchased from VWR (Fontenay sous Bois, France) at 20 mM and diluted in ultra pure RNAse-free water to obtain the solutions used in this study. EMA (phenanthridium, 3-amino-8-azido-5-ethyl-6-phenyl bromide) (Life Technologies) was dissolved in absolute ethanol to create the stock concentration of 5 mg / mL and then dissolved in ultra pure RNAse-free water to obtain the solutions used in this study. The EMA and PMA solutions were stored at −20°C in the dark. All the experiments with dyes were performed in light-transparent 1.5 mL microcentrifuge tubes (VWR).

### Binding of dyes to purified viral RNA

The effect of several EMA and PMA treatment processes on 10^8^ copies genome of viral RNA (RV, HAV) in 100 μL of phosphate-buffered saline (PBS) 1 ×, pH 7.0, were evaluated by testing several final dye concentrations (10, 20, 50, 100, 200 μM), with incubation of 2 h at 4°C in the dark and sample exposure to light for 15 min using the LED-Active® Blue system (IB - Applied Science, Barcelona, Spain). To determine whether PMA / EMA interfere with the ability of RT-qPCR to detect viruses, controls consisting of viral RNA that was treated with PMA / EMA without photoactivation were included with each dye concentration used. To attempt to remove the inhibitory effects of residual EMA / PMA on RT-qPCR, viral RNA treated with each dye concentration without photoactivation was purified using the QIAquick PCR purification kit (Qiagen, Courtaboeuf, France) according to the manufacturer’s instructions. Finally, to determine the efficiency of each concentration of PMA / EMA tested, treated viral RNA samples were subjected to photoactivation before the purification step using the QIAquick PCR purification kit. The negative control was a non-treated 1× PBS sample. The positive control was a non-treated viral RNA sample in 1× PBS. A non-treated viral RNA control sample was subjected to the photoactivation step to check the effect of the lamp. Finally, all these samples were subjected to RNA detection by RT-qPCR assays A. The experiments were performed three times for all viral RNA.

### Determination of the optimal dye concentration for viruses

The best dye (PMA / EMA) and its optimised concentration were determined for each viral target by testing five dye concentrations (5 μM, 20 μM, 50 μM, 75 μM, 100 μM). Briefly, in 100 μL of 1× PBS samples of 10^5^ TCID_50_ of RV (SA11), 10^3^ TCID_50_ of RV (Wa) and 6 × 10^4^ PFU of HAV were conserved at 4°C or inactivated at 80°C for 10 minutes. Next, samples were incubated with different dye concentrations for 2 h at 4°C in the dark and then exposed to light for 15 min using the LED-Active® Blue system. The negative control was an untreated 1× PBS sample. The positive controls were the non-dye-treated viral samples kept at 4°C or inactivated at 80°C for 10 minutes, used to calculate the reduction rates of the viral load. To check the effect of the lamp, the non-dye-treated viral samples kept at 4°C or inactivated at 80°C for 10 minutes and subjected to the photoactivation step were used as the controls. To check the effect of the dyes, the viral samples at 4°C or inactivated at 80°C for 10 minutes treated with 50 μM of dye without the photoactivation step were used as the controls.

Finally, all these samples were subjected to RNA extraction and detection by RT-qPCR assays A. The experiments were performed three times for each virus.

### Evaluation of the combined effect of dyes and surfactants

Tween 20 and IGEPAL CA-630 were purchased from Sigma-Aldrich (Saint-Quentin Fallavier, France) and Triton X-100 from Fisher Bioblock Scientific (Illkirch, France). These surfactants were dissolved in ultra pure RNAse-free water to obtain solutions at 1% and 10%. In 100 μL of 1× PBS, samples of 10^5^ TCID_50_ of RV (SA11), 10^3^ TCID_50_ of RV (Wa) and 6 × 10^4^ PFU of HAV were stored at 4°C or inactivated at 80°C for 10 minutes. The HAV and RV (Wa, SA11) samples were further treated with EMA 20 μM to which different final concentrations (0.1%, 0.5% and 1%) of the surfactants were added. The HAV and RV (SA11) samples were treated with PMA 50 μM to which different concentrations (0.1%, 0.5% and 1%) of the surfactants were added. The RV (Wa) samples were treated with PMA 75 μM to which different concentrations (0.1%, 0.5% and 1%) of the surfactants were added. Next, the samples were incubated for 2 h at 4°C in the dark and then exposed to light for 15 min using the LED-Active® Blue system.

The negative control was a non-inactivated and untreated 1× PBS sample. For the experiments at 4°C, the positive control was a non-inactivated and untreated virus sample incubated for 2 h at 4°C. For the experiments at 80°C, the positive control was an inactivated (10 min at 80°C) and untreated virus sample incubated for 2 h at 4°C. All non-inactivated samples and positive controls were subjected to infectious titration to check the effect of the surfactants on the infectious viruses. Finally, all these samples were subjected to RNA extraction and detection by RT-qPCR assays A. The experiments were performed three times for each virus. Concentrations of the surfactant (Tween 20, Triton ×100 and IGEPAL CA-630) added to the treated samples were applied to MA-104 cells in order to check their cytotoxicity (negative control). The experiments were performed three times for each virus.

### Evaluation of the incubation time with dyes and surfactants

The influence of the incubation time with dyes and surfactant were determined for HAV treated with EMA 20 μM + IGEPAL CA-630 0.5%, SA11 treated with PMA 50 μM and Wa treated with EMA 20 μM. Briefly, samples of 10^5^ TCID_50_ of RV (SA11), 10^3^ TCID_50_ of RV (Wa) and 6 × 10^4^ PFU of HAV were stored in 100 μL of 1× PBS at 4°C or inactivated at 80°C for 10 minutes and were further incubated with the corresponding selected dyes and surfactants for 30 min, 2 h and overnight in the dark and then exposed to light for 15 min using the LED-Active® Blue system.

The negative control was a non-inactivated and untreated 1× PBS sample incubated for 2 h at 4°C. For the experiments at 4°C, the positive control was a non-inactivated and untreated virus sample incubated for 2 h at 4°C. For the experiments at 80°C, the positive control was an inactivated (10 min at 80°C) and untreated virus sample incubated for 2 h at 4°C. Additional controls were performed to check the effect of the IGEPAL CA-630 0.5% alone on HAV regardless of the thermal inactivation and photoactivation. Finally, all these samples were subjected to RNA extraction and detection by RT-qPCR assays A. The experiments were performed three times for each virus.

### Thermal inactivation of viruses

Three series of HAV and RV strain (Wa, SA11) samples were inactivated thermally in 1× PBS by using a water bath set at 37°C and dry baths at 68°C, 72°C and 80°C. Aliquots of 50 μL of each virus were incubated for each temperature for 0, 1, 5, 10 and 20 min. Then, 150 μL of 1× PBS at 4°C were added to the samples and placed on ice. The negative control was a non-inactivated and untreated 1× PBS sample. The positive control was a non-inactivated and untreated virus sample stored at 4°C. Three 100 μL series of aliquots corresponding to 10^5^ TCID_50_ of RV (SA11), 10^3^ TCID_50_ of RV (Wa) and 6 × 10^4^ PFU of HAV were performed. The first series was kept to monitor loss of infectivity by performing virus titration on cells. The second series was subjected to direct RNA extraction. Finally, the third series was treated with selected dyes and surfactant. Typically, a final dye concentration of 20 μM of EMA and IGEPAL CA-630 0.5% were added to HAV aliquots, a final dye concentration of 20 μM EMA was added to RV (Wa) aliquots, and a final dye concentration of 50 μM of PMA was added to RV (SA11) aliquots. Then, all samples were incubated for 2 h at 4°C in the dark and then exposed to light for 15 min using the LED-Active® Blue system. After photo-activation, the virus samples were also subjected to nucleic acid extraction. Finally, RNA extracts obtained from the second and third series were quantified by testing the three RT-qPCR assays designed for each viral target. The experiments were performed three times for each virus.

### Viral RNA extraction

Nucleic acid extraction was performed in untreated virus samples and samples treated with dyes and surfactants. A hundred μL of the virus sample were supplemented with NucliSens® easyMAG™ lysis buffer (BioMérieux) up to 3 mL and subjected to the NucliSens® easyMAG™ platform for total nucleic acid extraction by the “off-board Specific A protocol” according to the manufacturer’s instructions. Lastly, nucleic acids were eluted in 70 μL of elution buffer and stored at −80°C.

### Primers and probes

Three RT-qPCR assays targeting the non-coding region at the 5’ end (5’-NCR) of HAV which have been described by Costafreda et al. [38], and adapted from Costafreda et al. [38] and Di Pasquale et al. [39,40] were used. The sequences of the primer pairs and the TaqMan probes used were as follows:

The HAV RT-qPCR assay A generates amplification products of 174 bp [38] and was recommended in the CEN/ISO/TS 15216 (qualitative / quantitative methods) for detection of HAV in foodstuffs. The sense primer (HAV68) was 5′-TCACCGCCGTTTGCCTAG-3′, the antisense primer (HAV241) was 5′-GGAGAGCCCTGGAAGAAAG-3′ and the TaqMan probe (HAV150 -) was 5′-FAM-CCTGAACCTGCAGGAATTAA–MGB-3′.

HAV RT-qPCR assay B generates amplification products of 353 bp. It exhibits the same sense primer and probe as HAV RT-qPCR model A associated with another antisense primer named HAV-399R: 5′ -GCCTAAGAGGTTTCACCCGTAG -3′ designed with Beacon Designer software.

Finally, the HAV RT-qPCR assay C adapted from Di Pasquale et al. [39,40] generates amplification products of 77 bp. The sense primer (HAVf ISS (459–478)) was 5′- GCGGCGGATATTGGTGAG**TT**-3′, the antisense primer (HAVr ISS (535–515)) was 5′- CAATGCATCCACTGGATGAGA-3′ and the TaqMan probe (HAVp ISS (484–511)) was 5′ ROX- Δ GACAAAAACCATTCAACGCCGGAG**GACT**-BHQ2-3′. When comparing to the model published by Di Pasquale et al. [39,40], “Δ” corresponds to a deletion of 4 nucleotides and the nucleotides in bold corresponds to insertions.

Three RT-qPCR assays targeting the rotaviruses were used. The RT-qPCR assay which has been described by Pang et al. [41] in the NSP3 region was used with a sense primer slightly modified with degenerated bases for matching with both human and simian strains.

Thus, RV RT-qPCR assay A generates amplification products of 87 bp. The sense primer (Rota NVP3-F) (positions: 963–982) was 5′-RYCATCTAYRCATRACCCTC-3′, the antisense primer (Rota NVP3-R) (positions 1034–1049) was 5′-GGTCACATAACGCCCC-3′ and the TaqMan probe (positions 984–1016) was 5′- FAM- ATGAGCACAATAGTTAAAAGCTAACACTGTCAA-BHQ1-3′.

RV RT-qPCR assay B generates amplification products of 313 bp. It exhibits the same antisense primer and probe as RV RT-qPCR assay A associated with another sense primer named Rota NSP3-736 F : 5′-GARTGGTATYTAAGATCWATGGAAT-3′ designed with Beacon Designer software.

RV RT-qPCR assay C designed in the NSP4 region with Beacon Designer software generates amplification products of 352 bp. The sense primer (rotaNSP4_166-188 F) was: 5′-ATTGCRYTGAAAACRTCAAAATG-3′, the antisense primer (rotaNSP4_517-493R) was: 5′-GCAGTCACTTCTYTTGGTTCATAAG-3′ and the TaqMan probe (rotaNSP4_486-462P) was 5′-ROX-YCCACTTTCCCAYTCTTCTAGCGTT-BHQ2-3′. Primers and probes were purchased from Eurofins (Les Ulis, France) and Applied Biosystems (Courtaboeuf, France).

### Real-time RT-qPCR conditions

One-step RT-qPCR amplifications were performed in duplicate on a CFX96™ real-time PCR detection system from Bio-Rad (Marnes-la-Coquette, France). Reactions were performed in a 25 μL reaction mixture containing 1× of thermoscript reaction mix, and 0.5 μL of Thermoscript Plus / Platinum Taq enzyme mix, which are components of the Platinum® Quantitative RT-PCR ThermoScript™ One-Step System (Fisher Bioblock Scientific, Illkirch, France), as well as 2 U RNAse inhibitor (Applied Biosystems), 5 μg of BSA (Ambion), 500 nM of forward primer, 900 nM of reverse primer, 250 nM of probe and 5 μL of RNA extract. The one-step RT-qPCR program was as follows: 60 min reverse transcription of RNA at 55°C, followed by a 15 min denaturation step at 95°C, and finally 45 cycles of 15 s at 95°C, 1 min at 60°C and 1 min at 65°C. The fluorescence was recorded at the end of the elongation steps (1 minute at 65°C) by the apparatus for each amplification cycle. Ct was defined as the PCR cycle at which the fluorescence intensity exceeded the threshold value. All samples were characterised by a corresponding Ct value. Negative samples gave no Ct value. A standard curve for each system was generated using 10-fold dilution of purified RNA. The slopes (*S*) of the regression lines were used to calculate the amplification efficiency (*E*) of the real-time qRT-PCR reactions, according to the formula: *E* = 10^|-1/*s*|^ -1 [42].

### Data analysis

The viral titers were obtained with cell culture assay and RT-qPCR according to the pre-treatment. Virus inactivation was determined by calculating the log_10_ (N_t_/N_0_), where N_0_ is the titre of the virus recovered on the positive control and N_t_ is the titre of the virus recovered on the tested sample.

Thermal inactivation kinetics were expressed as the virus survival ratio

(1) $$S_{i}\left(t\right)=\frac{N_{i}\left(t\right)}{N_{0}}$$

where N_i_(t) is the virus concentration measured with method *i* at time *t* and N_0_ is the virus concentration obtained by the RT-qPCR method.

GInaFiT, a freeware Add-in for Microsoft® Excel developed by Geeraerd et al. [43] was used to model inactivation kinetics. GInaFiT makes it possible to choose from different types of microbial survival models (nine) according to different statistical criteria (i.e., sum of squared errors, mean sum of squared errors and its root, R^2^, and adjusted R^2^). According to these criteria, the “log-linear + tail” inactivation model was found to be the most appropriate for describing inactivation curves regardless of the virus and the temperature of inactivation.

The log-linear + tail model can be expressed as followed:

(2) $$\log_{10}\left(S_{i}\left(t\right)\right)=\log_{10}\left(\left(S_{i,0}-S_{i,\mathrm{res}}\right)\cdot \exp\left(-k_{\max}\cdot t\right)+S_{i,\text{res}}\right)$$

where *k*_max_ (min^−1^), *S*_i,res_ and *S*_i,0_ are the model parameters.

*k*_max_ is the first order inactivation constant, i.e. it characterizes the slope of the linear decrease of concentration expressed as a logarithm. *k*_max_ is directly linked to the *D* value, the decimal reduction time, *k*_max_ = ln(10)/*D*. *S*_i,res_ characterizes the fraction of the population remaining constant in time, or, otherwise stated, not undergoing any significant subsequent inactivation regardless of the duration of the inactivation treatment. *S*_i,0_ is the initial survival ratio.

This ratio was expected to be equal to zero if the RT-qPCR method (*i* = 1) was used to quantify virus titer. *S*_i,0_ can also help to quantify the difference between RT-qPCR and pretreatment-RTqPCR (*i* = 2) or the cultural titration method (*i* = 3).

GInaFiT also returns the standard error values of the estimated parameter. These standard errors were used to construct asymptotic parameter confidence intervals. When no inactivation was observed, *k*_max_ and *S*_i,res_ were presented as zero with no confidence intervals, and the considered experiments were simply represented with *S*_i,0_. When no quantification was possible after 1 minute of treatment, corresponding to very fast inactivation, the limit of quantification (LOQ) value was used to set a value for *k*_max_ and *S*_i,res_. *k*_max_ was set at its minimum possible value, ln(10)·LOQ and *S*_i,res_ were set to their maximum possible value, i.e. LOQ. No confidence intervals were given for either parameter.

## Abbreviations

ATCC: American type culture collection; CEN: European committee for standardisation; dsRNA: Double-strand RNA; EMA: Ethidium monoazide; HAV: Hepatitis A virus; LOD: Limit of detection; LOQ: Limit of quantification; MEM: Minimum essential medium; NCR: Non-coding region; NoV: Norovirus; PBS: Phosphate-buffered saline; PCR: Polymerase chain reaction; PFU: Plaque-forming units; PMA: Propidium monoazide; RT-qPCR: Quantitative reverse transcriptase PCR; RV: Rotavirus; ssRNA: Single-strand RNA; TCID50: 50% tissue culture infectious dose.

## Competing interests

The authors declare that they have no competing interests.

## Authors’ contributions

CC and AF performed these experiments. LG performed statistical study. All authors wrote, read and approved the final manuscript.
